# Phytomediated-Assisted Preparation of Cerium Oxide Nanoparticles Using Plant Extracts and Assessment of Their Structural and Optical Properties

**DOI:** 10.3390/ijms24108917

**Published:** 2023-05-17

**Authors:** Nicusor Fifere, Anton Airinei, Florica Doroftei, Tudor Stefan Ardeleanu, Marius Dobromir, Daniel Tîmpu, Elena-Laura Ursu

**Affiliations:** 1 Petru Poni Institute of Macromolecular Chemistry, 41A Grigore Ghica Voda Alley, 700487 Iasi, Romania; fifere.nicusor@icmpp.ro (N.F.); florica.doroftei@icmpp.ro (F.D.); ardeleanu.tudor@icmpp.ro (T.S.A.); dtimpu@icmpp.ro (D.T.); ursu.laura@icmpp.ro (E.-L.U.); 2 Department of Exact and Natural Sciences, Institute of Interdisciplinary Research, Alexandru Ioan Cuza University of Iasi, 11 Carol I Blvd., 700506 Iasi, Romania; marius.dobromir@uaic.ro

**Keywords:** cerium oxide nanoparticles, plant extract, biogenic preparation, optical properties, XPS, Raman spectra, antioxidant activity

## Abstract

Cerium oxide nanoparticles were obtained using aqueous extracts of *Chelidonium majus* and *Viscum album*. X-ray diffractometry analysis confirmed the crystalline structure of the synthesized cerium oxide nanoparticles calcined at 600 °C. Scanning electron microscopy, UV-Vis reflectance and Raman spectroscopy, XPS, and fluorescence studies were utilized to interpret the morphological and optical properties of these nanoparticles. The STEM images revealed the spherical shape of the nanoparticles and that they were predominantly uniform in size. The optical band gap of our cerium nanoparticles was determined to be 3.3 and 3.0 eV from reflectance measurements using the Tauc plots. The nanoparticle sizes evaluated from the Raman band at 464 cm^−1^ due to the $F_{2g}$ mode of the cubic fluorite structure of cerium oxide are close to those determined from the XRD and STEM data. The fluorescence results showed emission bands at 425, 446, 467, and 480 nm. The electronic absorption spectra have exhibited an absorption band around 325 nm. The antioxidant potential of the cerium oxide nanoparticles was estimated by DPPH scavenging assay.

## 1. Introduction

Over the past few decades, there has been a significant development in the field of nanomaterials and nanotechnology. The interest in the utilization of nanomaterials, especially metal and metal oxide nanoparticles, has increased due to their unique characteristics, such as smaller particle size, large surface-to-volume ratio, and tunable morphological properties compared to their bulk counterparts. The nanoscale materials also possess different optical, thermal, chemical, magnetic, and mechanical properties relating to the bulk equivalents. As a result of these specific and tailorable properties, nanostructured materials have found applications in many fields, such as optics [1], environmental ones [2,3], catalysis [1,3], electronics [4], solar cells [5], biomedical [2,6], antibacterial ones [6,7], luminescent materials [8], etc.

Among the different kinds of nanoparticles, the metal oxide nanoparticles with tailored structures have gained great attention because of their unique size and their shape-dependent physico-chemical characteristics [9,10,11]. The design and synthesis of the metal oxide nanoparticles of controllable structural properties (optical, electrical, magnetic, catalytic, etc.) can be achieved through different fabrication conditions. In general, cerium can exist in two oxidation states: ${Ce}^{3+}$ and ${Ce}^{4+}$ due to the presence of ground state electrons in the 4f (Xe 4f^1^5d^1^6s^2^) orbital, which provide its redox properties [12,13]. At the nanoscale level, the cerium oxide presents a cubic fluorite structure, and the two ${Ce}^{3+}$ and ${Ce}^{4+}$ states can exist on the nanoparticle surface. The presence of the ${Ce}^{3+}$ state determines a charge deficiency, which can be compensated by a corresponding member of oxygen vacancies in the lattice. Therefore, the cerium oxide nanoparticles possess intrinsic oxygen defects. The activity and performance of materials containing cerium oxide nanoparticles depend on the content of the oxygen vacancies. In order to change the oxygen vacancy extent on the cerium oxide surface, many methods, such as decreasing nanoparticle size, morphology modification or metal doping, have been developed [14,15,16]. The properties of the cerium oxide nanoparticles depend on different parameters, such as synthesis procedures, type of surfactants, solvent or capping agents, and reaction temperature or time [8,17,18,19]. The synthetic strategies of the cerium oxide nanoparticles have been based mainly on physical and chemical methods. The physical methods for the cerium oxide nanoparticle preparation include pulsed laser ablation in liquids, the hydrothermal route, spray pyrolysis, chemical vaporization, ball milling, microwave irradiation, combustion, etc. [1,11,20,21,22,23]. The pulsed laser ablation technique presents the advantage of the lack of contamination with chemical reagents and a high stability of nanoparticles. The cerium oxide nanoparticles can be prepared via chemical pathways such as coprecipitation, sol-gel, microemulsion, electrochemical, and photochemistry, to name just a few [8,11,24,25,26,27]. The chemical and physical-mediated routes for the synthesis of the cerium oxide nanoparticles involve higher energy consumption to assure the temperature and pressure during synthesis, the use of the hazardous reagents and solvents generating toxic by-products for human health and the environment, long reaction time, or many steps in the synthesis protocol. In addition, some physical approaches need a high vacuum and also expensive equipment. However, these synthetic routes are not adequate when the nanoparticles are utilized in biomedical applications [28,29]. 

As a consequence, biological resources, such as plant extracts, fungi, algae, bacteria, or nutrients, become viable alternatives for synthetizing metal oxide nanoparticles, which are biodegradable and do not give off hazardous compounds [2,12,30]. The green chemistry methods mentioned above are eco-friendly, safer, and cost-effective processes for the preparation of the metal oxide nanoparticles. Among the biogenic methods, the plant-mediated synthesis of the cerium oxide nanoparticles using whole or parts of plants are very frequently utilized. The biogenic synthesis is similar to a chemical route where toxic and expensive reagents are replaced by plant extracts obtained from roots, leaves, flowers, seeds, fruits, or stems. The plant extracts contain different bioactive compounds such as flavonoids, polyphenols, terpenoids, amines, ketones, saponins, tannins, and other types of biomolecules, which are able to reduce the metal salt to nanostructured materials, acting as reducing and capping agents in the formation of metal oxide nanoparticles with different sizes and morphologies [16,26,30,31,32].

The biogenic synthesis of the cerium oxide nanoparticles have been applied already using different plant extracts from *Leuca aspera* leaves, *Gloriosa superba* L. leaves, *Morinda citrifolia* L. fruits, *Cydonia oblonga miller* seeds, *Rubia cordifolia* L. leaves, *Acorus calamus* rhizomes, and *Hibiscus sabdariffa* flowers, to name just a few [5,12,16,26,33,34,35,36]. The biogenic approach offers several benefits compared to the conventional methods, such as a minimum of hazardous by-products, without adding standard alkaline/acid compounds and without high temperatures.

The *Chelidonium majus* L. (Chelandine) is a familiar plant from Romania and it belongs to the *Papaveraceae* family. The plant develops on cultivated lands or close to houses. The biological active compounds can be found mainly in the roots, but also in flowers and leaves. The plant extract is a rich source of benzophenanthridine and isoquinoline alkaloids, such as chelerythrine, berberine, sanguinarine, coptsine, chelidonine, chelirubine, stylopsine, and methoxychelidonine. Other phytochemicals present in *C. majus* include organic acids (chelidonic, citric, malic, succinic), phenolic compounds (p-coumaric acid, ferulic acid, cafeic acid, kaemferol, rutoside, quercetol), phytosterols (stigmasterol, campesterol, sitosterol), saponosides, tannins, and mineral salts [37,38,39]. *Viscum album* extract was also used to prepare cerium oxide nanoparticles by the biogenic route. The phytochemical profile of *Viscum album* extract consists of lectines as the main group compounds, viscotoxins, polyphenolic compounds (chlorogenic, ferulic, p-coumaric, sinapic, and salicylic acids), phenylpropanoid derivatives (coniferin, syringenin, and lignans), flavonoids, terpenoid compounds, phytosterols, and carbohydrates [40,41]. These phytochemicals can replace the toxic reagents used in the reduction and capping processes in the formation of cerium oxide nanoparticles. In this way, the plant extract ingredients can stabilize the nanoparticle formation and monitor their morphology.

Here, the toxic effect of the utilized conventional chemicals in the synthesis of nanoparticles is reduced, and a more efficient synthetic strategy was developed in order to obtain cerium oxide nanoparticles with potential biomedical applications, including antioxidant, antibacterial, or antifungal activities. The cerium oxide nanoparticles prepared by biogenic routes are also biocompatible, which recommends them for use in the biomedical field.

In this study, the synthesis of the cerium oxide nanoparticles by a completely eco-friendly route, using aqueous extracts of *Chelidonium majus* (*C. majus*) roots and *Viscum album* (*V. album*) plants, has been reported. To the best of our knowledge, this study is the firstly report on *C. majus* extract-mediated synthesis of cerium oxide nanoparticles and their investigation by X-ray diffractometry (XRD), UV-Vis absorption, fluorescence and Raman spectra, scanning electron microscopy (SEM), and X-ray photoelectron spectroscopy (XPS). The prepared cerium oxide nanoparticles were explored for their antioxidant potential. However, *C. majus* extract was used previously for the preparation of the silver and zinc oxide nanoparticles [37,42]. 

## 2. Results and Discussion

**Figure 1:**
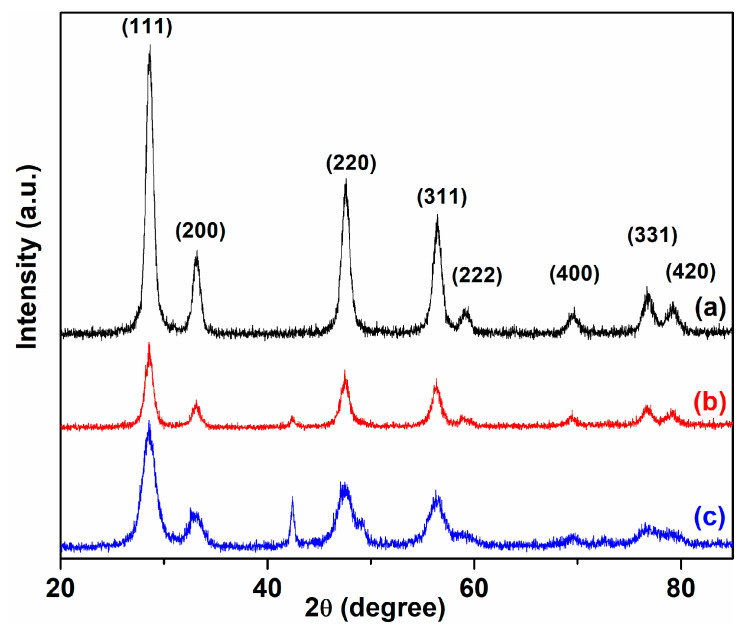
XRD pattern of cerium oxide nanoparticles using plant extract: (a) C-CM; (b) C-CM1; (c) C-VA.

Structural and crystalline characteristics of the cerium oxide nanoparticles obtained using *C. majus* plant extracts are investigated by XRD analysis. The XRD patterns of phytosynthesized cerium oxide nanoparticles are depicted in Figure 1. The X-ray diffraction peaks of C-CM are located at 2θ angles of 28.61°, 33.14°, 47.55°, 56.47°, 59.04°, 69.45°, 76.96°, and 79.08°, corresponding to (111), (200), (220), (311), (222), (400), (331), and (420) planes of the cerium oxide nanoparticles. This suggests a satisfactory agreement with the structure (space group Fm3m) of cerium oxide (JCPDS card No 34-0394), which indicates the high purity of the synthetized cerium oxide nanoparticles. These characteristic crystal planes in cerium oxide nanoparticles are consistent with those reported previously [8,19,26,43]. The diffraction pattern of the sample C-CM1 matched with that of sample C-CM (Figure 1). The corresponding Miller indices were provided, and no additional peaks were found. A highly intense diffraction peak of (111) provides the preferred orientation. 

The cell constant, *a*, was estimated using the following equation:

(1) $$a=\frac{\sqrt{3}\;\lambda }{2\;sin\theta }$$

where *λ* is the X-ray wavelength and *θ* is Bragg angle. The calculated value of the lattice parameter is 5.3765 Å for the C-CM sample, 5.3875 Å for the C-CM1 sample and 5.3980 Å for C-VA, respectively. The unit cell volume (V = a^3^) was found to be 155.417 ${Å}^{3}$ for C-CM, 156.373 ${Å}^{3}$ for C-CM1, and 157.341 ${Å}^{3}$ for C-VA (Table 1). The values of the lattice parameter of the as-synthetized samples are lower than the value corresponding to the bulk cerium oxide crystals (*a* = 5.412 Å). This can be due to the appearance of the oxygen vacancies leading to the modification of the Ce-O bonds, and, thus, a decrease in the lattice parameter and the crystallite size can occur. Lower values of lattice parameter *a* compared to the bulk value were reported in the literature for cerium oxide nanoparticles [34]. The crystallite size of the cerium oxide samples was determined using the Scherrer relation [44]:

(2) $$D=\frac{0.98\;\lambda }{\beta \;cos\theta }$$

where *β* denotes the full width at half maximum (FWHM), *λ* is the X-ray wavelength (*λ* = 0.15406 nm), and *θ* is the diffraction angle (in radians). The average crystallite sizes were found to be 10.14 nm and 9.86 nm for the cerium oxide nanoparticles prepared by *C. majus* plant extract and 5.97 nm for the C-VA sample, which matches with other published results, indicating the corresponding crystalline nature and the smaller grain size of the nanoparticles [10,45].

**Figure 2:**
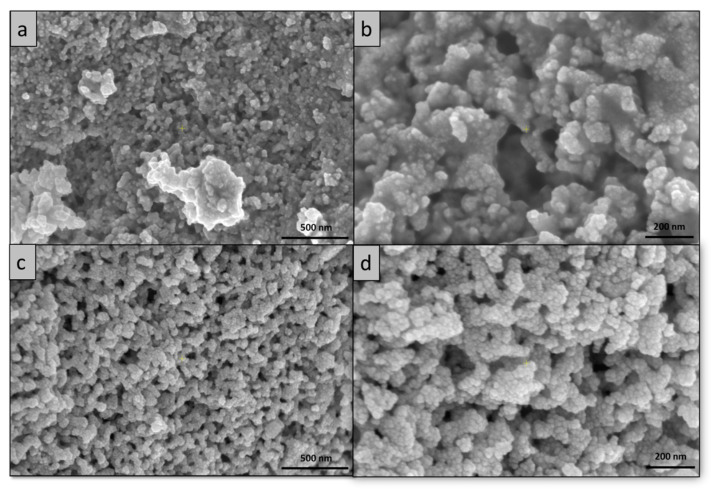
SEM morphology of synthetized nanoparticles: (**a**) C-CM1 50,000×; (**b**) C-CM1 100,000×; (**c**) C-CM 50,000×; (**d**) C-CM 100,000×.

The results of the SEM morphological analysis of the cerium nanoparticles are presented in Figure 2. As can be seen from the images, the nanoparticles have a spherical shape and they are homogeneous in terms of dimensions. In the case of the C-CM1 sample, the dimensions of particles are between 5–12 nm in size and appear as clusters of nanoparticles (Figure 2a). At higher magnification, it can be observed that the particles have an organic part with a smooth texture from the process of obtaining cerium nanoparticles (Figure 2b). C-CM nanoparticles also exhibit a spherical morphology with well-individualized particles dimensions in the range of 7–14 nm. 

**Figure 3:**
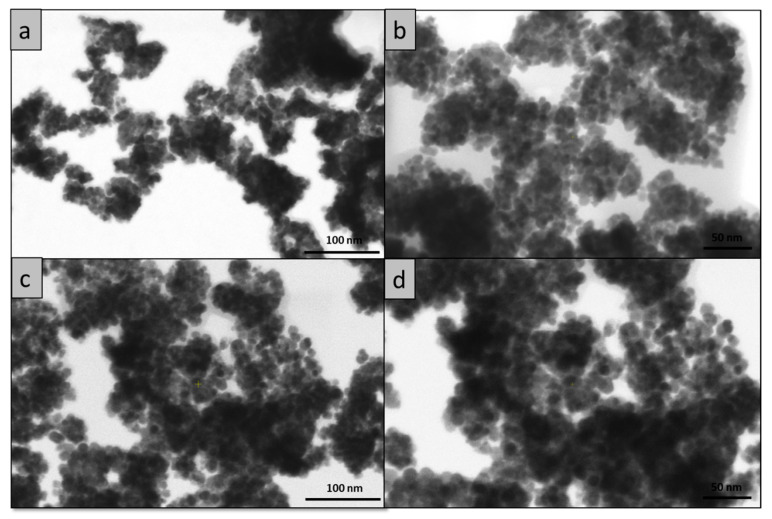
STEM morphology of cerium oxide nanoparticles: (**a**) C-CM1 250,000×; (**b**) C-CM1 350,000×; (**c**) C-CM 250,000×; (**d**) C-CM 350,000×.

To better identify the morphology of the synthesized cerium oxide nanoparticles, the samples were analyzed with a STEM3+ detector using the Verios G4 UC scanning electron microscope. The STEM mode in SEM allowed us to obtain images with a higher resolution, and for this reason more images were recorded in order to establish both the morphology and the dimensions of these nanoparticles. The STEM micrographs of the two analyzed samples are given in Figure 3 at two magnifications 250,000× (Figure 3a,c) and 350,000× (Figure 3b,d). From these images, it can be seen that the samples are homogeneous in terms of size and shape.

**Figure 4:**
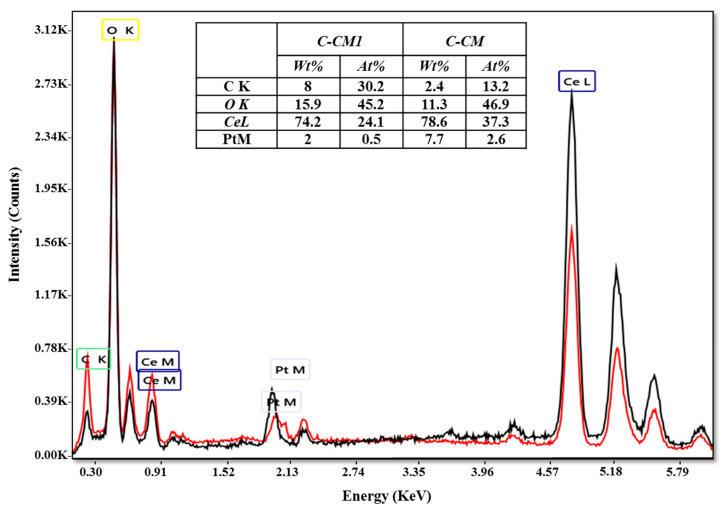
EDX spectra and chemical composition of the cerium oxide samples: C-CM (black color) and C-CM1 (red color).

EDX analysis was used to highlight the presence of the chemical elements of the synthesized samples, i.e., cerium (Ce) and oxygen (O). In Figure 4, the EDX spectra and the chemical composition are presented. Both spectra indicate the characteristic peaks of cerium (L- and M-shell transition energies) and oxygen (K-shell transition energy). From the EDX spectra, the presence of the C peak can be observed. In the case of the C-CM1 sample, the percent of C is higher compared to the C-CM sample. This was also evident in the case of the SEM micrographs, which can be attributed to the organic part of the synthesis mixture.

**Figure 5:**
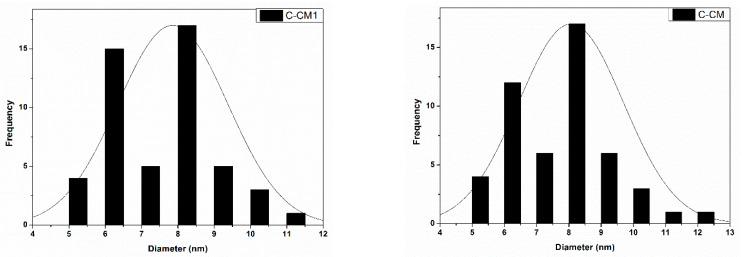
Particle size distribution of the cerium oxide samples determined from the STEM images.

In order to determine the average diameter of the obtained cerium oxide nanoparticles, a number of 50 nanoparticles were measured for each sample using the Image J software. The results are presented in the form of histograms in Figure 5. From the measurements, it transpired that in the case of the sample C-CM1, the average diameter of the particles was 8.2 nm, and in the case of the C-CM sample, the average diameter was 10.26 nm.

**Figure 6:**
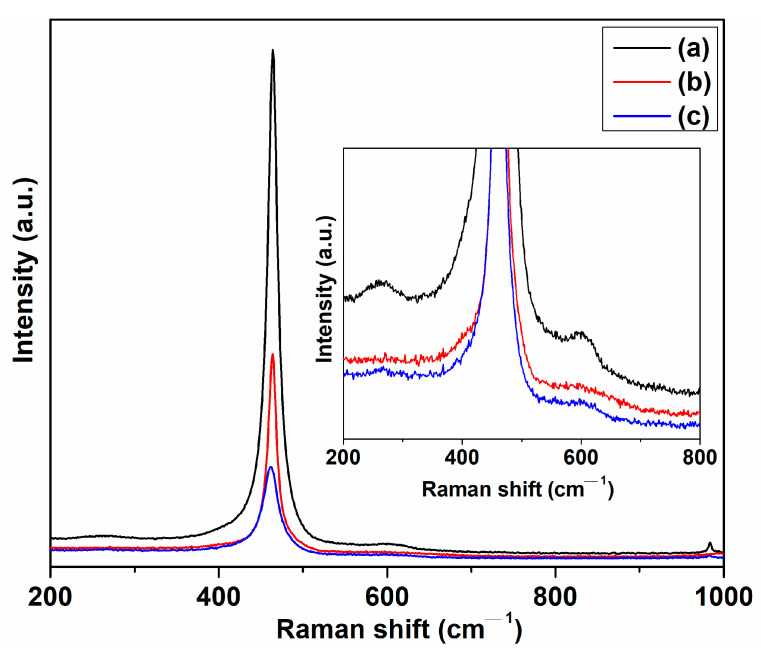
Raman spectra of cerium oxide nanostructures: (a) C-CM; (b) C-CM1; (c) C-VA.

The Raman spectra were also used to confirm the successful preparation of cerium oxide nanoparticles. Figure 6 shows the Raman spectra of cerium oxide nanostructures. For samples C-CM and C-CM1, a prominent Raman band has been observed at 463.9 and 463.6 cm^−1^, respectively, assigned to the Raman active $F_{2g}$ symmetrical stretching mode of the Ce-08 vibrational unit, indicating the cubic fluorite structure of cerium oxide nanoparticles [43,46,47]. The $F_{2g}$ stretching mode appears at 461.4 cm^–1^ for the C-VA sample. This observation confirms the XRD results. Furthermore, the cerium oxide-based samples obtained using plant extracts present two weak Raman modes around 601 and near 260 cm^−1^. The low intensity vibration mode at 601 cm^–1^ can be assigned to the oxygen vacancies in the cerium oxide lattice due to the presence of ${Ce}^{3+}$ ions, whereas the Raman band around 260 cm^−1^ can be due to the doubly degenerate transverse optical mode, contributing to the disorder in the system [26,45,47,48,49].

The particle size of cerium oxide has been estimated using the relation (3) [50]:

(3) $$\Gamma =5.48+98.4/D_{R}$$

where Γ in cm^−1^ represents the full width at half maximum of the Raman active mode, and *D_R_* stands as the particle size of the cerium oxide sample. According to relation (3), the particle size was found to be 9.69 nm for the C-CM sample, 12.09 nm for sample C-CM1, and 5.59 nm for C-VA, respectively. The particle size of the investigated cerium oxide samples practically coincided with those obtained from XRD and SEM data.

Using the grain size determined by relation (3), the average distance separating two lattice defects or the correlation length, *L*, can be estimated [51,52]:

(4) L=α2DR2DR−2α3+4DRα13

where *α* is the radius of the cerium oxide units (*α* = 0.34 nm) [51]. The defect concentration (N, cm^−3^) can be calculated by the relation (5) [50,52]:

(5) $$N=\frac{3}{4\pi L^{3}}$$

The values of *N* are given in Table 1. As can be seen, the sample C-VA having the lowest grain size exhibits a higher concentration of oxygen defect on the surface of the nanoparticles.

**Figure 7:**
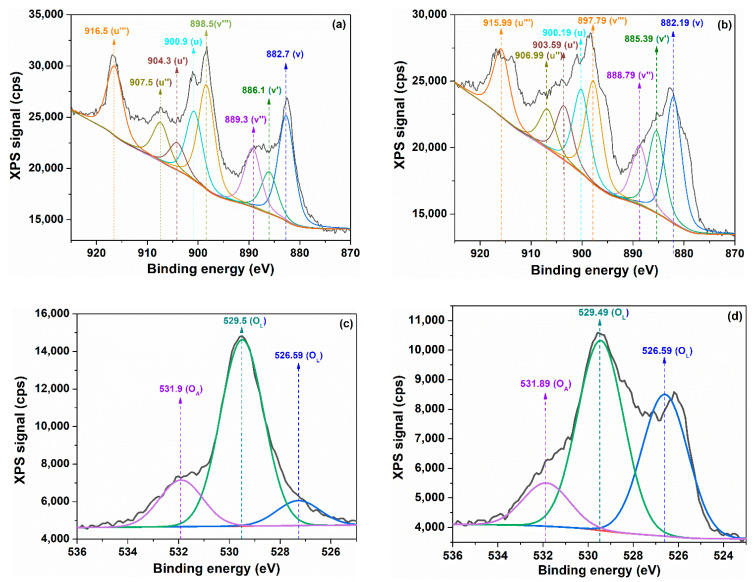
XPS Ce 3d (**a**) C-CM; (**b**) C-CM1 and O 1s (**c**) C-CM; (**d**) C-CM1 core levels spectra of cerium oxide nanoparticles.

The surface chemistry of cerium oxide nanoparticles, regarding to the composition and stoichiometry, was studied by X-ray photoelectron spectroscopy (XPS). The XPS core levels of Ce 3d and O 1s were performed and fitted by deconvolution in multiple Gaussian–Lorentzian peaks (Figure 7). It can see from Figure 7a,b that the Ce 3d core level spectra contain eight peaks, divided in two categories of V and U type, corresponding to the two states Ce ${3d}_{5/2}$ and Ce ${3d}_{3/2}$ [53]. These states are obtained by the splitting of the Ce 3d core level through spin–orbit interaction, and their peaks were grouped into four pairs: (U,V), (U′,V′), (U″,V″) and (U‴,V‴). According to the literature, the three doublets are characteristic of the ${Ce}^{4+}$ ion as U‴, U″; U, V‴; V″, V, and the peaks U′ and V′ are attributed to ${Ce}^{3+}$ [54,55]. The presence of the characteristic peaks for the ${Ce}^{3+}$ ion highlights the fact that cerium oxide nanoparticles are a mixture of cerium ions corresponding to the two valences +3 and +4. In order to calculate the content of ${Ce}^{3+}$ and ${Ce}^{4+}$, the sum of peak areas for each ion species against the sum of areas for whole Ce 3d spectrum was used as in the relations below [55,56]:

(6) $$\left[{Ce}^{3+}\right]=\frac{A_{U^{\prime }}+A_{V^{\prime }}}{A_{U^{\prime \prime \prime }}+A_{U^{\prime \prime }}+A_{U^{\prime }}+A_{U}+A_{V^{\prime \prime \prime }}+A_{V^{\prime \prime }}+A_{V^{\prime }}+A_{V}}\cdot 100$$

(7) $$\left[{Ce}^{4+}\right]=\frac{A_{U^{\prime \prime \prime }}+A_{U^{\prime \prime }}+A_{U}+A_{V^{\prime \prime \prime }}+A_{V^{\prime \prime }}+A_{V}}{A_{U^{\prime \prime \prime }}+A_{U^{\prime \prime }}+A_{U^{\prime }}+A_{U}+A_{V^{\prime \prime \prime }}+A_{V^{\prime \prime }}+A_{V^{\prime }}+A_{V}}\cdot 100$$

where *A_i_* represents the integrated area of peak “*i*”.

The values of the integrated areas for each sample are listed in Table 2, whereas the calculated values of the $\left[{Ce}^{3+}\right]$ and $\left[{Ce}^{3+}\right]/\left[{Ce}^{4+}\right]$ ratio with Equations (6) and (7) are reported in Table 3. As can be seen, sample C-CM1 has a higher $\left[{Ce}^{3+}\right]/\left[{Ce}^{4+}\right]$ ratio than sample C-CM. This indicates an alteration of the +4 pure oxidation number of cerium in nanoparticles such as ${CeO}_{2}$ with the generation of mixed oxide species as ${CeO}_{x}$ where ${{Ce}_{2}O}_{3}$ was formed. The increase in the content of Ce^3+^ can appear due to the reduction of the oxidation state of cerium from +4 to +3 as a result of the elimination of oxygen during the synthesis process, as in the reaction below:

(8) $${CeO}_{2}\overset{}{\to }{CeO}_{x}+\left(1-\frac{x}{2}\right)O_{2}$$

The ${Ce}^{3+}$ ion can contribute to the formation of ${{Ce}_{2}O}_{3}$ that coexists with ${CeO}_{2}$ to form mixed ${CeO}_{x}$ oxides with 1.5 < *x* < 2. This happens if we assume that the whole O content is the sum of the required oxygen to fully oxidize cerium ions with both positive oxidation numbers, ${Ce}^{3+}$ (*x* = 3/2) and ${Ce}^{4+}$ (*x* = 2). Taking into consideration the above assumption, the stoichiometric parameter x can be calculated using Equation (9) [57]:

(9) $$x=\frac{3}{2}\left[{Ce}^{3+}\right]+2\left[{Ce}^{4+}\right]$$

It can be observed from Table 3 that the values of *x*, calculated with Equation (9), are slightly lower than two, and so the coexistence of ${CeO}_{2}$ with ${{Ce}_{2}O}_{3}$ is possible, but with a clear majority of cerium oxide with the upper oxidation number due to the fact that $\left[{Ce}^{4+}\right]\gg \left[{Ce}^{3+}\right]$. Based on these considerations, the ${CeO}_{x}$ mixed oxides may have a slightly altered fluorite cubic structure. This was not clearly observed in the XRD diffractograms, which leads us to the conclusion that the amount of ${{Ce}_{2}O}_{3}$ is somewhat negligible. Because the content of ${Ce}^{3+}$ for both samples cannot be considered negligible, it can be assumed that the oxygen deficiency in cerium oxide is mainly determined by oxygen vacancies and, to a small extent, by the presence of ${{Ce}_{2}O}_{3}$ oxide. The oxygen vacancies can appear due to the necessity of compensation of the charge imbalance generated by the presence of the ${Ce}^{3+}$ ion, as dopant in nanoparticles, but with the fluorite cubic crystal structure preserved. 

Equation (10) gives the oxygen content, taking into account the Ce 3d spectrum only, and, therefore, the calculated values for *x* can be considered as having a pronounced theoretical character. The actual stoichiometry, *x’*, can be directly calculated as the ratio of the sums of integrated areas of XPS peaks of the O 1s (A_O_) and Ce 3d ($A_{Ce}$) cores, divided by the ratio of the sensibility factors of O ($S_{O}$ = 0.711) and Ce ($S_{Ce}$ = 7.399) atoms as follows [57]:

(10) $$x^{\prime }=\frac{A_{O}}{A_{Ce}}\cdot \frac{S_{Ce}}{S_{O}}$$

The O 1s spectra of cerium oxide nanoparticles consist of three peaks, which can be associated with the different types of oxygen species bound to cerium having different oxidation numbers. Generally, in the literature, the peaks with a lower energy level, in our case 526.59 eV and 529.50 eV for C-CM or 526.59 eV and 529.49 for the C-CM1 sample, are attributed to lattice oxygen noted $O_{L}$, and the peaks corresponding to a higher energy level such as 531.89 eV for C-CM and 531.89 eV for C-CM1 are assigned to weakly adsorbed oxygen species on the surface of nanoceria, noted $O_{A}$ [58,59].

Table 3 illustrates the values of the actual stoichiometry *x’* calculated with relation (10), taking into account the integration areas of the peaks from the O1s spectra. These values are clearly lower than two for both samples. The sample C-CM has the real stoichiometry, *x’*, lower than the sample C-CM1, indicating a greater oxygen deficiency in the case of the first sample. Moreover, from the same table, it is observed that the real stoichiometry parameter *x’* is lower than the theoretical one, *x*, for both samples. In other words, the oxygen content of nanoparticles is considerably lower than that required for the complete oxidation of cerium atoms to ${Ce}^{3+}$ and ${Ce}^{4+}$ ions. The positive value of difference *∆x = x − x’* indicates the presence of oxygen vacancies due to a lower oxygen amount than the stoichiometric needs. The *∆x* value corresponding to sample C-CM is higher than that corresponding to C-CM1, despite the higher content of ${Ce}^{3+}$ of the latter. Therefore, increasing the content of ${Ce}^{3+}$, depending on the synthesis process, the possibility of ${{Ce}_{2}O}_{3}$ formation to the detriment of oxygen vacancies increases. Thus, sample C-CM, with a lower content of ${Ce}^{3+}$, as compared to C-CM1, generates a small enough charge imbalance so that it can be compensated by oxygen deficiencies. When the ${Ce}^{3+}$ content increases, in the case of the C-CM1 sample, the charge imbalance can no longer be effectively compensated by the oxygen vacancies, so a larger amount of Ce(III) oxides is formed.

**Figure 8:**
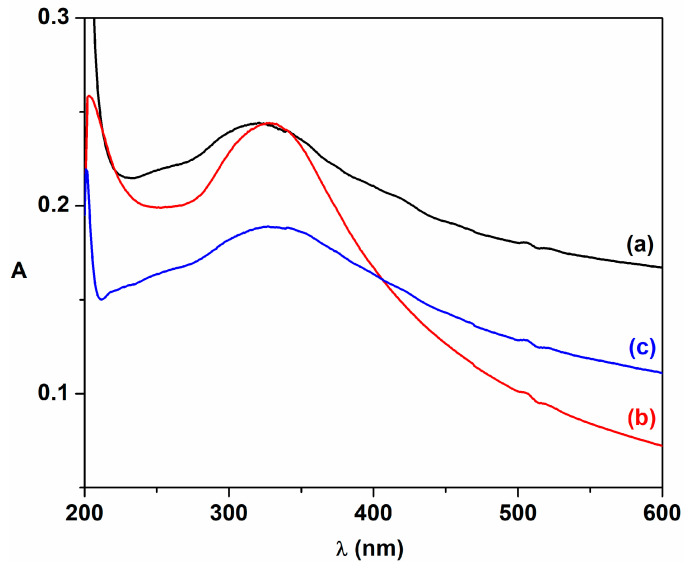
UV-Vis absorption spectra of cerium oxide nanoparticles: (a) C-CM; (b) C-CM1; (c) C-VA.

The UV-Vis absorption spectra of cerium oxide nanoparticles dispersed in isopropanol that were obtained by the biogenic approach using plant extracts are shown in Figure 8. These samples display an absorption band around 330 nm, which can be caused by the direct charge transfer transition from the valence band of O 2p to the 4f conduction band of ${Ce}^{4+}$ ions [60,61,62,63]. Because these materials absorb the UV radiation below 300 nm, the obtained cerium oxide nanoparticles could be used as UV-shielding materials [5].

**Figure 9:**
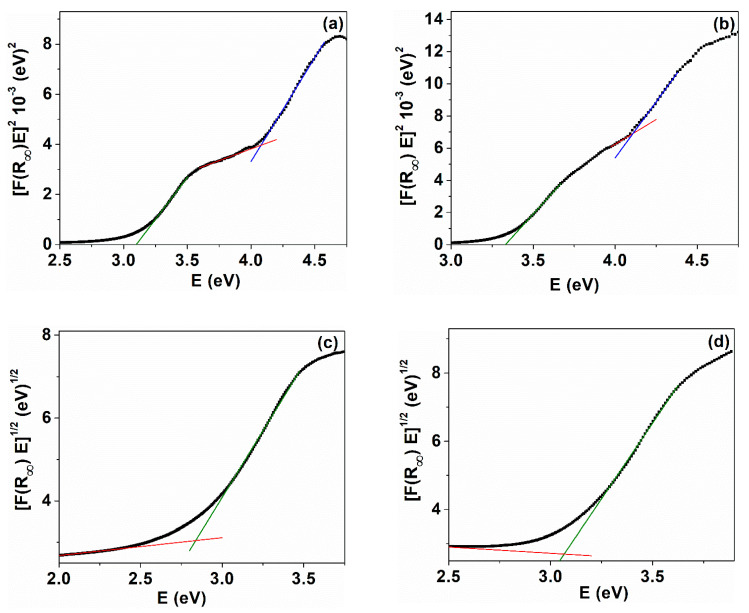
Variation of [F(R)hν] versus hν = E for cerium oxide nanoparticles: *n* = 1/2 (**a**) C-CM; (**b**) C-CM1; *n* = 2 (**c**) C-CM; (**d**) C-CM1.

The band gap energy is an important characteristic of the nanomaterials that can be used in optoelectronics. The band gap of the synthesized samples was estimated from diffuse reflectance spectra, applying the Tauc relation in which the absorption $F\left(R_{\infty }\right)$ is related to the reflectance by the Kubelka–Munk equation $F\left(R_{\infty }\right)=\left(1-R_{\infty }^{2}\right)/\left(2R_{\infty }\right)$, where R represents the sample reflectance and $F\left(R_{\infty }\right)$ is also named the Kubelka–Munk function. In these conditions, Tauc relations can be written as [64]: FR∞hν1n=Ahν−Eg, where h is the Planck constant, ν denotes the frequency of the incident photon, A is a constant for material, n is 1/2 for a direct transition or 2 for an indirect transition, and $E_{g}$ is the hand gap energy. By plotting FR∞hν1n as a function of hν, the band gap energy values were obtained for the two transitions of the cerium oxide nanoparticles. The linear part of the curves was extrapolated to intercept the photon energy axis in order to obtain $E_{g}$ [65]. Our results indicate that from the Kubelka–Munk plots, two band gap energies for the direct transition (Figure 9a,b) and one band gap for the indirect transition (Figure 9c,d) were observed for samples C-CM and C-CM1, respectively. This dual band gap behavior for the direct transition was also reported for cerium oxide nanoparticles [8,66]. The two-step transition in the band gap was shown by Ho et al. for rod- and spherical-shaped cerium oxide nanoparticles [66]. A band gap value of 3.10 eV and 3.33 eV ($E_{g}^{d1}$) was found for the direct transition of samples C-CM and C-CM1. For sample C-CM, the $E_{g}$ is smaller than the band gap value corresponding to bulk cerium oxide (3.19 eV) [67,68]. This red shift in the band gap can be due to the presence of higher number of defects, leading to the formation of some localized states within the band gap of the materials [48,69]. Although the nanoparticle size of the two samples practically does not differ between them, the band gap of sample C-CM1 is greater compared to C-CM due to the lower defect content (Table 3). The indirect band gap energies are lower than the direct ones. The calculated values of indirect band gap were estimated from Kubelka–Munk plots as being: 2.83 eV (C-CM) and 3.07 eV (C-CM1) (Figure 9c,d). The values of $E_{g}^{d}$ and $E_{g}^{i}$ of the cerium oxide nanoparticles are consistent with some reported results [67,70].

**Figure 10:**
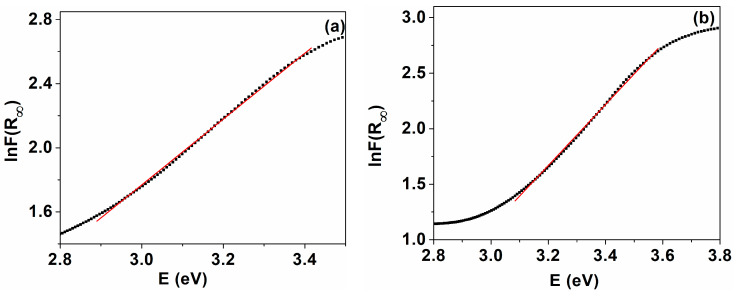
Urbach plots of cerium oxide nanoparticles: (**a**) C-CM; (**b**) C-CM1.

The Urbach energy ($E_{U}$) near the optical band edge of the cerium oxide nanoparticles was determined in order to obtain information about the influence of the lattice defects on the band structure and the contribution of these defects in the localized states of the band gap. The Urbach energy has been estimated from the empirical relation [71,72]:

(11) $$\alpha =\alpha_{0}\;exp\left(h\nu /E_{U}\right)$$

where $\alpha_{0}$ a constant, *hν* is the incident photons energy, α represents the absorption coefficient, and $E_{U}$ denotes the Urbach energy describing the width of localized state in the band gap. In this case, the absorption coefficient is proportional to $F\left(R_{\infty }\right)$, and equation then becomes by linearization:

(12) $$lnF\left(R_{\infty }\right)=ln\beta +h\nu /E_{U}$$

where *β* represents a constant. Urbach energy was determined as the inverse slope of the linear fit resulting from the plot of $lnF\left(R_{\infty }\right)$ as a function of hν (Figure 10). The Urbach energy for the cerium oxide nanoparticles was found to be 486.7 meV for C-CM and 364.1 meV for C-CM1, respectively. The increase in the band tail energy ($E_{U}$) in sample C-CM shows an increased degree of the structural disorder, defect states, and vacancy level. This fact is confirmed by XPS data (Table 3).

The relative position of the valence band (VB) maximum and the conduction band (CB) potentials of the cerium oxide nanoparticles can be estimated using the optical band gap energy values according to the following relations [32,73]:

(13) $$E_{VB}=\chi -E_{C}+0.5E_{g}$$

(14) $$E_{VB}=\chi -E_{C}-0.5E_{g}$$

where χ represents the electronegativity of cerium oxide (5.56 eV) and $E_{C}$ is the energy of free electrons on the hydrogen scale (−4.5 eV) [60,74].

The calculated values of CB were −0.53, −0.65 and −0.60 eV, respectively, whereas the following values for CV were found: 2.57, 2.69, and 2.64 eV for C-CM, C-CM1, and C-VA samples, respectively. The results show that the highest values for $E_{VB}$ and $E_{CB}$ were found out for samples the C-CM1 and C-CM samples, respectively. The CB and VB values of the cerium oxide nanoparticles were in good agreement with those previously reported [73,75].

The refraction index of the cerium oxide nanoparticles can be determined using the relation [76]:

(15) n2−1/n2+2=1−Eg/2012

The estimated values of the refractive index of our samples were 2.37 for C-CM and 2.15 for C-CM1, respectively. These low refractive indices determine high transmittance in the visible range and high absorption in the UV region.

**Figure 11:**
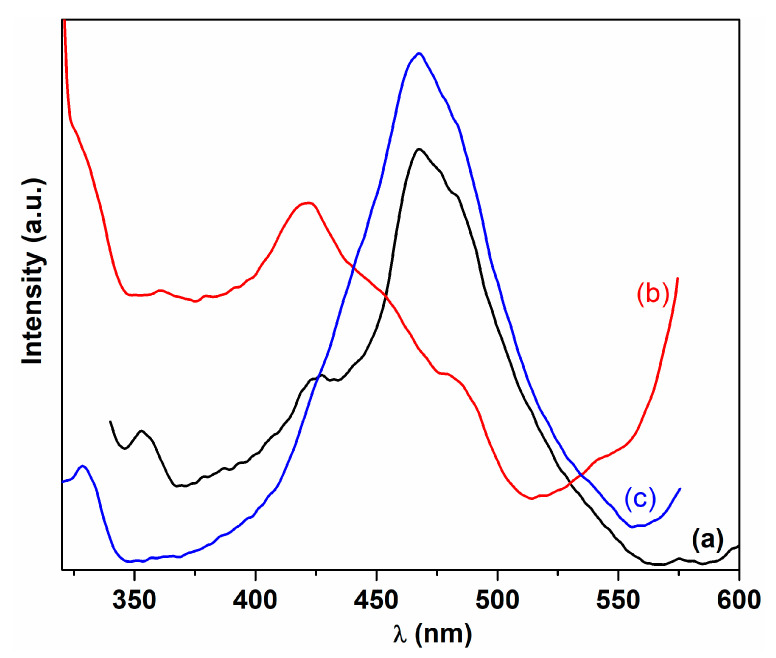
Emission spectra of cerium oxide nanoparticles: (a) C-CM; (b) C-CM1; (c) C-VA.

Figure 11 displays the emission spectra of cerium nanoparticles at the excitation wavelength of 300 nm. The emission spectra of cerium oxide samples dispersed in isopropanol show a broad band profile ranging from violet to green. The fluorescence spectra of samples C-CM and C-VA have similar characteristics (Figure 11). It can be seen that a strong blue emission band positioned at 468 nm with a blue-green shoulder at 481 nm and a blue band of lower intensity at 427 nm were observed. The sample C-CM1 displays a different emission pattern, which consists of an emission band at 422 nm, being the most intense band, a blue emission band (shoulder) at about 446 nm, and a blue-green emission band at 480 nm (Figure 11). These emission bands are consistent with data already reported in the literature for cerium oxide nanoparticles [19,77,78,79]. The emission energies of cerium oxide nanoparticles are revealed to be below the band gap energy due to the presence of the crystal defects or the oxygen vacancies in the crystal lattice. The cerium oxide samples prepared using *C. majus* extract have different fluorescence profiles, probably due to the formation of different densities of defects in crystal lattice during synthesis of the samples. Generally, the emission bands in the range of 350–550 nm could be attributed to the surface structural defects in the cerium oxide nanoparticles, including oxygen vacancies with trapped electrons that are localized between the Ce 4f state and the O 2p valence band in lattice [16,19,77,80]. The blue emission bands located around 422 nm (C-CM1) and 427.5 nm (C-CM) can be associated with defect states existing between Ce 4f level and O 2p valence band, such as dislocations, which is useful for the fast oxygen transportation [4,34,48,81]. The prominent blue emission band around 467 nm and the blue-green shoulder around 480 nm can be assigned to oxygen vacancies and oxygen interstitial defects in the crystalline unit with defect states below Ce 4f level [10,77,81].

The room temperature fluorescence spectra of as-prepared cerium oxygen nanoparticle using *C. majus* extract were excited at different wavelengths (270, 280, 300, 320, and 360 nm). The emission spectra of the samples are practically identical, but the band intensities are different, and as the excitation wavelength increases, a lower emission response was recorded. In our case, a defect concentration of 1.61 × 10^21^ cm^–3^ was obtained for sample C-VA, which exhibits higher emission intensity.

The investigation of the antiradical scavenging properties of the synthetized cerium oxide nanoparticles was made using the reduction reaction of the DPPH colored free radical. In a very general mechanism, the free radical can accept an electron, donated from cerium oxide nanoparticles through redox-cycling between +3 and +4 oxidation states. As the reaction advances, the color saturation decreases due to the disappearance of the DPPH free radical with the increase in the contact time. The complete reduction of DPPH by cerium oxide leads to a discoloration of the DPPH and nanoparticle mixture solution from purple to pale yellow or even to colourless. This process can be monitored by recording the absorbance decay at 515 nm and calculating the antioxidant activity or radical inhibition rate (AA%) with the following formula:

(16) $$AA\%=\left(1-\frac{A_{sample}-A_{blank}}{A_{ref}}\right)\cdot 100$$

where $A_{blank}$ and $A_{ref}$ represent the absorbance of the sample, blank, and reference solutions, respectively. The sample is represented by the cerium oxide nanoparticle suspension in methanol solution of DPPH. The blank and reference solution represent the nanoparticle suspension and the DPPH solution, respectively, and separately, in methanol, with the same concentration as in the sample.

**Figure 12:**
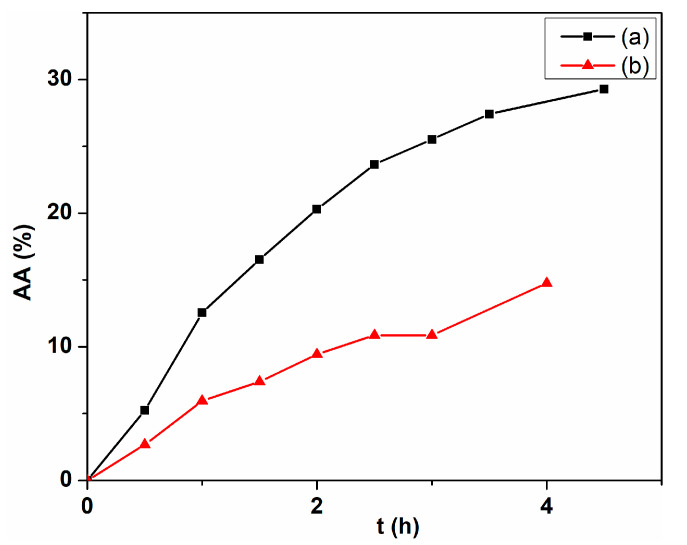
Antioxidant potential of cerium oxide nanoparticles: (a) C-CM; (b) C-CM1.

The scavenging effect was studied for the biogenic synthesized samples, C-CM and C-CM1, by DPPH colorimetric assay, and the results are illustrated in Figure 12. The absorbance decay at 515 nm was rather slow for both samples with a radical inhibition rate around 28.5% for C-CM sample and 14.8% for C-CM1, respectively. Our last studies on the cerium oxide nanoparticles, obtained by precipitation reaction with inorganic bases as the precipitating agent, proved that the oxygen deficient sites are the active sites that enhance the possibility of ${Ce}^{3+}$ to donate an electron [82]. This process transforms the cerium oxide nanoparticles into a reducing agent. However, these nanoparticles were both deficient and rich in oxygen [8]. In the present study, the cerium oxide nanoparticles were obtained using natural extracts, and both samples are oxygen deficient, as can be seen from the XPS data. However, it is useful to note that sample C-CM1, which has a higher oxygen deficiency than C-CM, presents a lower inhibitory activity than the last sample. Instead, the antioxidant activity is very well correlated with the concentration of ${Ce}^{3+}$ ions, also calculated from the XPS data (Table 3). The C-CM sample has a considerably higher concentration of ${Ce}^{3+}$ ions than C-CM1, so the antioxidant activity is in directly proportional relationship with superficial ${Ce}^{3+}$. This is logical, taking into account that ${Ce}^{3+}$ ion has the ability to reduce to ${Ce}^{4+}$, yielding an electron to an oxidizing agent. The results are in agreement with the data in the literature, where the difference of ${Ce}^{3+}$ content is determined by the difference due to the nanoparticle dimensions [83,84]. Smaller nanoparticles means a higher specific surface and, therefore, a higher surface concentration of ${Ce}^{3+}$ ions occurs, leading to a higher scavenging effect. As a result, the antioxidant activity can be determined by the dual effect of the ${Ce}^{3+}$ concentration and the nanoparticle dimension. In our case, the samples C-CM and C-CM1 have almost the same dimension (Table 1). Therefore, in this case, the scavenging effect is clearly correlated with the concentration of ${Ce}^{3+}$, independent of the size of the nanoparticle. As can be seen, the antioxidant effect is not satisfactorily high and, therefore, further efforts will have to be made to increase it by keeping the optimal ratio between the two oxidation states of cerium.

## 3. Materials and Methods

### 3.1. Materials

Cerium (III) nitrate hexahydrate (${Ce\left({NO}_{3}\right)}_{3}\cdot 6H_{2}O$) was purchased from Sigma Aldrich, Darmstadt, Germany. 2,2-Diphenyl-1-picrylhydrazyl (DPPH), isopropanol, methanol and acetone were procured from Merck.

### 3.2. Preparation of Chelidonium majus Plant Extract

The *Chelidonium majus* roots were collected from Iasi county, Romania and washed with running water, and then with double distilled water before cutting and drying in dark at room temperature for 5 days. After that, they were crushed to obtain a fine powder. 10 g of *C. majus* powder was then added to 100 mL of double distilled water under continuous magnetic stirring for 2 h at 50 °C. The prepared extract was filtered using Fisher Whatman-540 filter paper. The filtrate was stored at 5 °C in a refrigerator for further use.

### 3.3. Synthesis of Cerium Oxide Nanoparticles Using C. majus Extract 

Briefly, 7 g of cerium nitrate hexahydrate was dissolved in 10 mL of double distilled water. This solution was added drop-by-drop into 80 mL of aqueous *C. majus* extract at 50 °C under vigorous stirring for 50 min. The color change from the white product to the yellowish precipitate at a high reaction time indicates the formation of cerium oxide nanoparticles. This reaction mixture resulted in the yield of a yellow color precipitate. Once the reaction was finished, the resulting solution was allowed to stand at 5 °C for 24 h to decant. The precipitate was then subjected to centrifugation at 6000 rpm for 45 min at 5 °C in order to remove any residuals. The resulting cerium oxide nanoparticles were dried in a hot oven at 60 °C for 24 h. The dried material was ground into fine powder and calcined for 5 h at 600 °C. This cerium oxide sample was designated as C-CM. The aqueous solution obtained after filtration was diluted with acetone in the volume ratio of 1:10. The resulting precipitate was centrifuged (6000 rpm) at 5 °C to obtain a yellow product. The sample was washed twice with double distilled water and then dried in a conventional furnace at 60 °C for 24 h, followed by calcination at 600 °C for 5 h. Thus, the cerium oxide nanopowder denoted as C-CM1 was obtained.

### 3.4. Preparation of Viscum Album Aqueous Extract

Fresh *Viscum album* plant was rinsed with running water followed by bi-distilled water several times at room temperature for 7 days, and then ground to fine powder. Aqueous extraction was made by heating the powdered material (10 g) in bi-distilled water (100 mL) at 70 °C for 5 h on a magnetic stirrer hotplate. The extract was filtered using Fisher Whatmann-540 filter paper and deposited at 5 °C. 

### 3.5. Biogenic Synthesis of Cerium Oxide Nanoparticles Using Viscum Album Extract (C-VA)

Firstly, 4.6 g of (${Ce\left({NO}_{3}\right)}_{3}\cdot 6H_{2}O$) was dissolved in 10 mL of bidistilled water. The above solution was then added dropwise to 85 mL of *V. album* extract. This reaction mixture was stirred vigorously for 20 min at 50 °C. The resulting precipitate was stored for 24 h at 5 °C. Further, the collected precipitate was centrifuged (6000 rpm) for 45 min and washed with ethanol. The resulting product was calcined in air at 600 °C for 5 h, after which it was allowed to cool in an oven at room temperature, and was then ground to obtain a fine powder of cerium oxide nanoparticles.

### 3.6. Characterization

#### 3.6.1. X-ray Diffractometry

An X-ray diffraction (XRD) pattern of the cerium oxide nanoparticles was performed on a Bruker 18 Avance X-ray diffractometer using the CuKα irradiation line (λ = 1.5406 Å), between the scan angles 10° and 90° with an accelerating voltage of 40 kV and a current of 40 mA. The values of the full width at half maximum (FWHM) and the diffraction angles (θ) were utilized to estimate the crystallite size and the peak broadening. 

#### 3.6.2. Spectral Measurements

The Raman analysis of our samples was made with a micro-Raman system (Renishaw in a Via Reflex) using a He-Ne laser (New Mills, UK), the beam having wavelength of 633 nm. The Raman determinations were performed at atmospheric pressure and room temperature. The electronic absorption spectra are monitored using SPECORD 210Plus spectrometer (Analytik Jena, Jena, Germany) in isopropanol. The UV-Vis diffuse reflectance spectra were measured using Shimadzu UV-3600 spectrometer equipped with an integrating sphere within a wavelength range of 300–800 nm at room temperature. Photoluminescence measurements were performed on a Perkin Elmer L55 luminescence spectrometer in 10 mm path length quartz cells in isopropanol. X-ray photoelectron spectroscopy (XPS) determinations had been performed on Physical Electronic PHI-500 Versa Probe instrument using a monochromatic AlKα radiation source (1486.6 eV). Survey scan spectra were recorded on the ranges 870–930 eV and 522–538 eV, respectively. 

#### 3.6.3. SEM Investigations

The morphology and elemental composition of the synthesized cerium oxide samples were analyzed with a Verios G4 UC Scanning electron microscope (Thermo Scientific, Brno, Czech Republic) equipped with energy dispersive X-ray (EDX) analyzer (Octane Elect Super SDD detector, Pleasanton, CA, USA). The atomic and weight percentages of elements presented in the samples were estimated using a EDX spectra. For SEM analysis, the samples were fixed on aluminum stubs with double-adhesive carbon tape and coated with 6 nm platinum using a Leica EM ACE200 Sputter coater (Vienn, Austria) to provide electrical conductivity and to prevent charge buildup during exposure to the electron beam. SEM investigations were made in immersion mode using a secondary electron detector (Through the Lens Detector, TLD) at an accelerating voltage of 5 kV. Scanning transmission electron microscopy (STEM) studies were performed using the STEM3+ detector (Bright-Field Mode) at an accelerating voltage of 30 kV. For STEM analysis, the samples were dispersed in water and ultrasonicated, and were then placed on carbon-coated copper grids with a 300-mesh size and dried in an oven until the solvent was removed. The size distribution of the nanoparticles was calculated using ImageJ software based on a representative set of STEM images taken from different areas on the samples.

## 4. Conclusions

Cerium oxide nanoparticles were successfully prepared by adopting a facile approach using aqueous extract of abundant available *C. majus* and *V. album* plants. All the samples show absorption bands below 400 nm according to the electronic absorption spectra, suggesting the formation of the cerium oxide nanoparticles. The cerium oxide nanoparticles present almost spherical-shaped morphology, having a cubic fluorite structure with the average particle size ranging from 6 to 10 nm according to XRD data, confirmed by SEM analysis (8–10 nm) and Raman results due to the existence of the strong Raman band around 464 cm^−1^. Band gaps were determined from the diffuse reflectance spectra using Tauc plots. The photoluminescence spectra confirmed the presence of localized states in the gap by the blue and blue green emissions. Using the same precursors in the synthesis of the cerium nanoparticles, two fractions were obtained—the first by primary precipitation (C-CM), and the second by reprecipitation of the supernatant (C-CM1). The sample C-CM revealed a ${Ce}^{3+}$ content of 13.49%, leading to a higher oxygen deficiency (Δ*x* = 0.37) than sample C-CM1 (Δ*x* = 0.67) with a higher level of ${Ce}^{3+}$ at 22.16%. These findings have shown that the *C. majus* plant can act as a stabilizing agent for obtaining nanoparticles. The presence of oxygen vacancies in the crystal lattice of cerium oxide nanoparticles is closely related to their antioxidant activity, and C-CM has an antioxidant activity value of 28.5%, almost double that of the C-CM1 sample. This paper has attempted to report the biogenic preparation of the cerium oxide nanoparticles using toxic-free solvents in order to reduce environmental pollution.

## Figures and Tables

**Table 1:** Structural parameters of cerium oxide nanoparticles.

Sample	λ_max_ (nm)	D_XRD_ (nm)	a (Å)	ν (cm^−1^)	D_R_ (nm)	V (Å^3^)	N (cm^−3^)
C-CM	321	10.14	5.3803	463.9	9.69	155.74	9.03 × 10^20^
C-CM1	328	9.86	5.3875	463.6	12.09	156.37	7.17 × 10^20^
C-VA	327	5.97	5.4023	461.4	5.59	157.66	1.61 × 10^21^

**Table 2:** Integrated areas of Ce 3d and O 1s XPS peaks.

Sample	Ce 3d_3/2_				Ce 3d_5/2_				O 1s		
	U‴	U″	U′	U	V‴	V″	V′	V	O_A_	O_L1_	O_L2_
C-CM1	24,513	14,310	19,634	30,158	37,141	21,682	29,749	45,694	3740	16,433	12,263
C-CM	32,269	18,315	12,929	32,362	48,892	27,750	19,589	49,033	5288	21,166	2832

**Table 3:** Concentrations of ${Ce}^{3+}$ and ${Ce}^{4+}$ ions and stoichiometry of cerium oxide nanoparticles.

Sample	$\left[{Ce}^{3+}\right]$ (%)	$\left[{Ce}^{4+}\right]$ (%)	$\left[{Ce}^{3+}\right]/\left[{Ce}^{4+}\right]$	*x*	*x′*	Δ*x*
C-CM1	22.16	77.84	0.28	1.89	1.51	0.37
C-CM	13.49	86.51	0.16	1.93	1.26	0.67

## Data Availability

Data presented in this study are available upon reasonable request from the corresponding author.
